# The Association for Human Pharmacology in the Pharmaceutical Industry London Meeting 2018: Brexit and Other Challenges in Early Phase Drug Development

**DOI:** 10.3389/fphar.2018.01301

**Published:** 2018-11-19

**Authors:** Susan Reijntjes, Muna Albayaty, James Bush, Joseph Cheriyan, Anthea Cromie, Annelize Koch, Michael Hammond, Stuart Mair, Peter Scholes, Ulrike Lorch, Steffan Stringer, Jorg Taubel, Timothy C. Hardman

**Affiliations:** ^1^Niche Science & Technology Ltd., Richmond, United Kingdom; ^2^Parexel International, Northwick Park Hospital, London, United Kingdom; ^3^Covance Clinical Research Unit Ltd., Leeds, United Kingdom; ^4^Cambridge University Hospital NHS Foundation Trust, Cambridge, United Kingdom; ^5^Celerion, Belfast, United Kingdom; ^6^Simbec-Orion Group, Slough, United Kingdom; ^7^Clinical Quality Management Solutions, Oxford, United Kingdom; ^8^Quotient Sciences, Nottingham, United Kingdom; ^9^Richmond Pharmacology Ltd., London, United Kingdom; ^10^Alwyn Consulting, Guildford, United Kingdom

**Keywords:** early phase clinical drug development, meeting report, association for human pharmacology in the pharmaceutical industry, regulatory, brexit

## Abstract

The Association for Human Pharmacology in the Pharmaceutical Industry (AHPPI) annual meeting focused on the changing face of early phase drug development and opened with a keynote speech concerning the revolution in pharmaceutical medicine over the last 30 years and the impact this has had on the way patients are treated. Examples were presented of how translational pharmaceutics is being used to tackle the high drug candidate failure rate and is improving productivity when moving drug candidates from the laboratory through to clinical proof of concept. The European Medicines Agency revised 2007 Risk Mitigation guideline on first in human (FIH) clinical trials was discussed. The focus of the revised guideline, which came into force in February 2018, is on risk mitigation and promotion of safety and will assist drug sponsors with the design and performance of early clinical studies. The use of integrated adaptive protocol designs in early clinical development was discussed in relation to the challenges involved when running early phase clinical trials in patients. The Health Regulatory Authority presented its strategies to ensure that following Brexit, the United Kingdom remains an attractive place to conduct Phase I clinical trials. The Medicines and Healthcare products Regulatory Agency confirmed that in the event of a “no deal” Brexit, it is well placed to implement and influence many provisions of the new EU CTR. The meeting provided an opportunity to discuss the changing regulatory environment and the opportunities and challenges facing the United Kingdom following Brexit with invited speakers from a range of disciplines including drug development, clinical trials and research organizations, government science policy and regulatory agencies.

## Introduction

The Association for Human Pharmacology in the Pharmaceutical Industry (AHPPI)^1^ is the oldest not-for-profit professional association involved in early phase drug development in the world. Founded in 1988, it provides a forum for continuing education in clinical pharmacology and in the regulatory aspects of early development of new medicines. The AHPPI’s 1-day annual meeting held in London on June 22, 2018 focused on the challenges of early phase drug development in 2018 and strategies employed by the pharmaceutical industry to address them. By bringing together stakeholders from a range of disciplines including drug development, clinical imaging, clinical trials and research organizations, government science policy and clinical trials regulation, the AHPPI committee ensured this would provide engaging, wide-ranging and balanced viewpoints from professionals within the pharmaceutical industry. This report summarizes the key findings derived from the meeting.

## Keynote: the Past, Present and Future of Pharmaceutical Medicine and Its Impact on How We Treat Patients

Professor Alan Boyd (President of the Faculty of Pharmaceutical Medicine of the Royal College of Physicians. United Kingdom) spoke about the technical revolution in pharmaceutical medicine over the 30 years since the AHPPI was founded, and the impact it has had on the way we treat patients.

In 1988, monoclonal antibody therapies had just begun to receive United States Food and Drug Administration (FDA) approval, statins and antiretroviral therapies for the treatment of human immunodeficiency virus were still under development, the human genome project was underway and cell and gene therapies and immunotherapies were a distant dream.

Fast-forward to 2018, and approximately 30% of available medicines are biologically based, 10 cell and gene therapies have been approved in Europe and immunotherapies are being successfully used in oncology and rare diseases. Sequencing of the human genome has led to the development of the field of pharmacogenomics and the advent of personalized medicine. Today, more than 100 FDA-approved drugs carry pharmacogenomics information in their labels in therapy areas as diverse as analgesics, antivirals and anticancer therapeutics (NIH National Human Genome Research Institute, 2017).

Technology is changing how disease is diagnosed and treated. For example, the FDA has granted the first approval of a digital medicine system. Abilify MyCite^®^ (aripiprazole tablets that incorporate a digital sensor, Proteus Digital Health, 2017) records when patients take their medication and is approved for use in the treatment of schizophrenia, bipolar disorder and depression (Peters-Strickland et al., 2018). The pill sends a signal to a wearable patch that transmits the information to a mobile device, such as a phone. Patients can track ingestion of the medication and their physician can access dosing data through a web-based portal.

We have seen the rise of the patient voice. Increasingly, patients have been searching, finding and using internet health information to become more informed about their disease states, and challenging the types of care being offered. Online patient communities and patient-led charities funding research programs have also emerged. In response to this, the United Kingdom Medicines and Healthcare products Regulatory Agency (MHRA) has set up the Patient Group Consultative Forum with the goal of establishing patient voice within the agency.

Professor Boyd spoke about the future challenge of treating an increasingly elderly population, in particular, polypharmacy. And yet, most medicines development is conducted in younger subjects looking at single agents. Nearly 50% of older adults are taking one or more medications that are most likely not medically necessary (Maher et al., 2014). The absolute clinical benefit to the individual of each additional medicine is expected to reduce when a patient takes multiple preventative medicines whereas the risk of harm increases.

Today, doctors are navigating an array of high technology, complex ethical considerations, and shifting patient expectations. The General Medical Council, working in partnership with organizations involved in medical education and training in the United Kingdom, is updating its postgraduate medical training curriculum in order to train healthcare professionals for the future.

The way patients are treated is changing, driven by a revolution in biologics, technology and digital medicines. With more collaboration between medical professionals, academics and the pharmaceutical industry, Professor Boyd concluded that the future for pharmaceutical medicine is looking very bright.

## Integrating Adaptive Pharmaceutical and Clinical Strategies to Improve Flexibility and Efficiency in Early-Phase Drug Development

Professor Peter Scholes (Chief Scientific Officer, Quotient Sciences, United Kingdom) described how increasing costs and high drug candidate failure rates have generated interest in integrated adaptive protocol designs, which combine healthy volunteer, first-in-human (FIH) studies with multiple ascending dose studies and patient studies to obtain early efficacy data. However, these protocols alone do not address the high drug candidate failure rate. Poor oral bioavailability and tissue exposure remain key contributors in approximately a third of drug-failure cases. While the protocols themselves may be integrated and adaptive, a degree of flexibility in the drug product would be required to address drug delivery challenges.

Considerable investment is required to manufacture multiple drug product dose strengths in quantities required for clinical trials in early drug development. Different suppliers may be required for drug substance formulation development, clinical trial manufacturing and packaging. If a candidate fails toxicology assessments or proves suboptimal after dosing, the drug developer must start the process all over again, stalling clinical development.

Translational pharmaceutics is a unique platform integrating formulation development, real-time, adaptive Good Manufacturing Practice (GMP) manufacturing and clinical testing offering shorter timelines, reduced costs and increased success rates in early phase drug development. One major advantage of translational pharmaceutics is that the drug product dose and formulation can be adapted in real-time in response to emerging clinical safety, pharmacokinetic, and pharmacodynamic data. Modified drug products can be manufactured and administered during the study, and the clinical data evaluated anew. By repeating this cycle as frequently as every 14 days, clinical data can be used to adjust the formulation between each study period so that by the end of the protocol the potential to have met the clinical endpoints has been maximised and the drug product is optimized for progression to the later stages of development. Implementing this 14-day “make-test” cycle can reduce a typical formulation development timeline by 6 months or more.

Professor Scholes presented examples of the successful use of translational pharmaceutics. The first demonstrated a tailored, adaptive approach to early phase drug development. A FIH study in healthy volunteers was conducted to assess an oral phosphoinositide 3-kinase inhibitor. Three capsule formulations (powder blend, lipid suspension, and spray dried dispersion) were developed and prioritized for evaluation. The single ascending dose arm of the study was used to investigate precise dose adjustments. Levels of target inhibition were assessed and a formulation suitable for patient trials was identified. Using this approach, the time from initiating formulation development activities for the FIH study through to oncology patient supply was only 12 months.

The challenge in a second example was to effectively deliver the selective ionotropic glutamate receptor 5 antagonist, LY545694, to a preferred absorption region of the upper gastrointestinal tract, and thus achieve a pharmacokinetic profile similar to a reference controlled release (CR) formulation with a reduced dose. Complicating factors were that LY545694 had a short half-life, caused dose-limiting adverse events and could only be absorbed through the small intestine. A new CR formulation prototype with variable drug release rates was developed by adjusting the polymer composition of the tablet, the dose was lowered and the formulation radiolabeled to support scintigraphic imaging to visualize and quantify *in vivo* tablet performance. A crossover study was conducted in healthy volunteers and the formulation was assessed using emerging clinical data; interim decisions on its performance were based on pharmacokinetic and scintigraphic imaging data (Lobo et al., 2012). Two more formulation prototypes were assessed using the 14-day “make-test” cycle. Efficient delivery of LY545694 to the site of absorption resulted in 30% higher bioavailability and an appropriate formulation prototype was selected for further development. The programme took less than 8 months to complete.

The final case study focused on a challenging proof-of-concept study conducted in an orphan pediatric patient population. Real-time adaptive GMP manufacturing provided an on-demand, personalized drug product supply to more than 180 patients in multiple sites across eight countries.

Professor Scholes concluded that translational pharmaceutics provides the opportunity to incorporate both adaptive protocols and adaptive drug products into clinical trial designs to enable better informed decision making that is also fast and cost-effective. More than 80% of studies supported by Quotient Sciences from 2012 to 2017 have benefited from translational pharmaceutics.

## Medical Imaging in Early Phase Clinical Trials

Dr. Philip Murphy (Head, Clinical Imaging at GlaxoSmithkline, GSK, United Kingdom) talked about the potential of medical imaging as a platform supporting drug development.

Although imaging plays an important role across all phases of drug development, Phase I trials incorporating state of the art imaging technologies can enable early characterization of biodistribution, target engagement and pharmacology. Several medical imaging techniques are currently available for use in early phase drug development. High-resolution computed tomography can measure structural changes, e.g., the volumetric reduction of a tumor during an oncology disease response assessment. Downstream markers of pharmacology can be studied using functional techniques such as magnetic resonance imaging, e.g., in rheumatoid arthritis, volumes of inflamed synovial membrane can be quantified from gadolinium contrast enhancement imaging. Positron emission tomography (PET) provides a highly sensitive means to track radiolabelled drugs or probe molecules to determine tissue distribution. Existing methods can be integrated into clinical studies, although efforts need to be made to standardize data acquisition and quality, particularly in multi-centre studies, which are recognised as having high variability in measurement (Matthews et al., 2012). Where new methods need to be developed, planning should give careful consideration to time needed to create a robust technique that can be used in the study.

Despite the progress made in the development and availability of medical imaging techniques, their practical value has been limited. Many structural techniques provide little insight into underlying pathophysiologies. More informative techniques are too costly to move into the clinic or lack validation, giving them little value beyond measuring exploratory endpoints. The result is that only a small proportion of potential medical imaging toolsets translate into worthwhile drug development tools.

There are many ways that the United Kingdom pharmaceutical industry can support the development and application of medical imaging technology innovation for clinical trials. These could include optimizing the interface between the pharmaceutical industry and external networks in academia, commercial diagnostics and contract research organizations; more pre-competitive method development activities across the industry; creating a network of centres with standardised technologies; training a new generation of imaging scientists; and ensuring that medical imaging technologies have a clear path toward decision making.

## The Impact of the Revised European Medicines Agency First-In-Human Guidelines on Toxicity Assessments and Dose Escalation

David Jones (Expert Pharmaco-Toxicologist, Clinical Trials Unit at the MHRA, United Kingdom) talked about the European Medicines Agency (EMA) revised 2007 Risk Mitigation guideline on FIH clinical trials (CHMP, 2017). The focus of the revised guideline, which came into force on 1^st^ February 2018, is on risk mitigation and promotion of safety and will assist sponsors with the design and performance of early clinical studies of a new investigational medicinal product (IMP) in humans. The guideline extends the existing European Union (EU) guidance to address FIH and early phase clinical trials with integrated adaptive protocols. The first edition of the EMA guideline followed the devastating events that occurred during the FIH study of TGN1412 in March 2006. The first administered dose of this CD28 superagonistic antibody induced cytokine release syndrome in the healthy volunteers in the study.

The 2017 revisions were considered necessary following review of the BIAL clinical trial incident in France in 2016 in which a healthy volunteer died. The compound being tested was BIA 10-2474, a fatty acid amide hydrolase inhibitor that enhances endogenous endocannabinoid concentrations. Although BIA 10-2474 had not been administered to humans, fatty acid amide hydrolase inhibitors had been studied previously in a number of clinical trials. Although other compounds of the class had generally failed to show therapeutic effects across various indications, no safety concerns had been raised. The protocol is available (BIAL-Portela and Ca. SA-ANSM, 2015) and it is apparent that pharmacology data with BIA 10-2474 were limited and no information was provided on how the no-observed-adverse-effect-level (NOAEL) had been calculated.

The updated guideline has not called for an increase in the amount of non-clinical data required for supporting first-in-human clinical trials, but has emphasized the critical value of pharmacology and understanding the mode of action of an IMP.

The updated guideline describes strategies to mitigate and manage risks for clinical trial participants. Drug exposure should be estimated for the initial dose and, following dose escalations, remain within a predefined maximum exposure limit. The starting dose for healthy volunteers should be below the pharmacologically active dose, unless a robust justification can be made for a higher dose. Similar considerations should apply to the identification of a safe starting dose in patients. The starting dose and a maximum level of exposure (usually based on NOAEL), as well as dose escalation steps, should be justified and outlined in the protocol. Dose escalation should be guided by the dose/exposure-toxicity or the dose/exposure-effect relationship defined in the non-clinical studies, and adapted following review of emerging clinical data from previous subject cohorts.

Guidance is now also provided on the use of integrated adaptive protocols as this approach requires information generated in previous parts of the trial to be analyzed and integrated into an assessment in a limited timeframe before making a decision on proceeding to the next part. The updated guideline provides recommendations for stopping criteria, and guidance on the rolling review of emerging data. There is also guidance on the handling of adverse events in relation to stopping rules, and information on how to progress to the next dosing level. The selection of an appropriate dosing interval and duration of dosing should consider the specific pharmacokinetic and pharmacodynamic characteristics of the IMP, the available non-clinical safety data and all data obtained from subjects in previous single dose cohorts. Particular attention should be paid to linear versus non-linear pharmacokinetics in the expected concentration range, the pharmacokinetic IMP profiles in the expected concentration range, the pharmacokinetic half-life versus duration of action, and the potential for drug accumulation. Multiple dosing parts can explore different dosing regimens and schedules, such as a moving from once-daily dosing to twice-daily dosing. A maximum duration of dosing should now be stated in the protocol for every cohort. The expected exposure after multiple dosing should have been covered during preceding single ascending dose parts of a study. However, if emerging clinical data following multiple dosing suggests tolerance to adverse effects seen in a single ascending dose part of a study, higher exposures in a multiple ascending dose part can be considered, provided this option is pre-specified and below the set maximum exposure, or is submitted as a substantial amendment to the protocol.

Dr. Jones noted an apparent perception that effects related to primary pharmacology should not be considered adverse. In his opinion, as long as this belief persists, there will always be the potential for another tragedy similar to the BIAL trial, particularly as qualitative and quantitative differences may exist in biological responses to a new IMP between animals and humans.

Finally, Dr. Jones reminded the audience that the MHRA can provide scientific and regulatory advice during any stage of the initial development of an IMP. Scientific advice can also be obtained from the Committee for Medicinal Products for Human Use and the sole remit of the Scientific Advice Working Party is to provide scientific advice and protocol assistance to applicants.

## Challenges of Running Early Clinical Trials in Patients

Charlotte Chadwick (Head of the MAC Research Early Phase Unit, United Kingdom) discussed her experiences in running early phase clinical trials. Twenty years ago all Phase I studies were conducted in healthy volunteers, included special populations (e.g., smokers, elderly, or post-menopausal women) and investigated one primary objective. Over the past 10 years, the increasing popularity of integrated adaptive protocols has seen more Phase 1 studies that include a patient cohort or are conducted entirely in patients. Since MAC Research opened in 2016, 80% of their Phase I clinical trials have involved patients.

Stringent inclusion/exclusion criteria limiting co-morbidities and the use of concomitant medications mean recruiting a particular patient population is more challenging than recruiting healthy volunteers. Also, the increasing length of clinical trials means that generally only elderly patients can commit the time to participate. Consequently, it takes time and energy to obtain sufficient patient numbers to participate in a Phase I study.

Ethical and regulatory challenges also exist when including patients in Phase I studies. Patients may gain no benefit if they receive placebo and they could be exposed to an IMP that excludes them from future potentially beneficial studies, such as monoclonal antibodies. Many Phase I patient clinical trials are run by the National Health Service (NHS) and although clinical trials in healthy volunteers conducted in Phase I units adhere to strict regulatory guidelines, this is not a requirement for patient clinical trials run by the NHS. In addition, The Over-volunteering Prevention System (Boyce et al., 2013), run by the Health Research Authority (HRA) to protect volunteers in clinical trials, is not used by the NHS.

Including patients in Phase I studies can present significant operational challenges. Depending on the therapeutic area, these may include the necessity for specialist physicians, support staff and equipment, longer trial timelines (as it takes longer to recruit patients than healthy volunteers), arranging in-patient stays and repeated follow-ups. A robust on-site system is necessary to house and dispense concomitant medications and to ensure patients do not “swap” their medications. There is also the risk that patients with the same condition will talk to each other and “learn” new disease symptoms. Although Phase I clinical trials conducted in healthy volunteers provide information on safety, pharmacokinetics and tolerability of an IMP, they do not provide information on patient dose, but this type of study in patients is long and time consuming.

Ms. Chadwick described a successful clinical trial conducted in patients with migraine that circumvented the requirement to recruit large numbers (Mac Clinical Research, 2018). It is difficult to predict when a “natural” migraine will occur, therefore, glyceryl trinitrate (GTN) was used to induce a migraine-like attack in 20 patients who had a history of naturally occurring migraine. The study demonstrated that delayed headache following GTN exposure in migraineurs was a reasonable surrogate for spontaneous migraine attacks; it was reproducible, migrainous in character and treatable. This technique could be used to rapidly assess the potential efficacy of novel compounds under development for the treatment of migraine using modest numbers of patients.

## Capitalizing on Our Experience in the Review of Early Phase Clinical Trials – Phase I Issues From the Health Research Authority Perspective

Catherine Blewett (Health Research Authority, HRA, Improvement and Liaison Manager, United Kingdom) discussed issues related to obtaining approval for Phase I clinical trials through the HRA and Research Ethics Committee (REC) review process. Health Research Authority approval is required for any research project involving recruitment of patients via the NHS in England and Wales. The approval process includes assessment of governance and legal compliance by the HRA, and independent ethical opinion provided by a REC.

The HRA has been listening to applicants to discover what is most important to them when submitting a Clinical Trials of an Investigational Medicinal Product (CTIMP) application. The most important request is for short and predictable CTIMP approval timelines. Once a CTIMP has been submitted and applicants have received their ethical review validation letter, they are then required to attend either a proportionate review (if the clinical trial raises no material ethical issues) or a full REC meeting. Applicants are notified of the REC’s decision, usually within 10 working days, and receive one of the following decisions: favorable opinion, favorable opinion with additional condition, provisional opinion or an unfavorable opinion. The quickest route for HRA approval is to receive a favorable opinion after the initial review (received by 12.5% of applications in 2017).

The HRA want more applicants to receive a favorable opinion upon their first application, so they have audited their previous responses. They observed that the most frequent reason for a provisional rather than a favorable opinion was poorly written patient information sheets, in which details on dose escalation, sample size or subject recruitment strategy were considered to be unclear. Reasons for unfavorable opinions most frequently centered on incomplete or insufficient pre-clinical data. The HRA have also developed guidance for staff and committee members on how to approach requests for application changes so that they can be addressed as a condition rather than a provision, therefore avoiding further REC review.

Ethics committees generally consist of volunteers who perform reviews on a broad range of research topics. Challenges faced by committee members include tight decision timelines, lack of knowledge of innovations that invoke different ethical issues, and understanding complex clinical trial designs. Committee members come from different social backgrounds and do not always have the same ethical opinions. To address these challenges, the HRA are conducting ethical debates with REC members to identify areas of inconsistency or differing opinions.

The HRA aims to modify the CTIMP approval process to reduce time from submission to recruitment of first participant. In addition, it recognizes that integrated design clinical trials are a time and cost effective way to run trials and is supportive of this approach. Along with the MHRA, the HRA is part of a collaborative working group contributing to a consensus paper, which will provide guidance on innovative clinical trial designs, including integrated adaptive designs. The HRA’s aim is to make the United Kingdom globally competitive with a world-class governance infrastructure, ensuring it remains an attractive location to conduct clinical trials.

## United Kingdom Clinical Trial Regulation: Challenges and Opportunities of the New Clinical Trial Regulation

Sam Bunce (PhD student, Leeds University and former intern at the Parliamentary Office of Science and Technology [POST] Houses of Parliament, United Kingdom) explained that POST is Parliament’s in-house source of independent, balanced and accessible analysis of public policy issues related to science and technology. He presented their research that focused on the United Kingdom regulatory landscape as viewed by stakeholders, the impact of the current EU Clinical Trials Directive (CTD) 2001/20/EC, the aims of the new EU Clinical Trials Regulation (CTR) (EU No. 536/2014) and the new shared EU-wide portal and database (European Commission, 2014), and the challenges and opportunities for the United Kingdom clinical trials industry post-Brexit.

In 2013, in response to the declining number of trials being conducted in the United Kingdom, which fell by 22% between 2007 and 2011, a House of Commons Select Committee inquiry into clinical trials concluded that the complex approval process and the 2001 EU CTD were instrumental in creating barriers to conduct clinical trials in the United Kingdom. In response to their findings, the HRA now provides a single approval process for all ethical permissions. Generally, stakeholders from across the pharmaceutical industry felt that the United Kingdom regulators, including the HRA and the MHRA, provided a (relatively) streamlined process for approving clinical trials and took a lead in driving innovative practice within the United Kingdom. Their overall impression of the CTD, a directive that aimed to harmonize clinical trial regulation, increase patient safety and make running multi-center clinical trials across EU Member States easier, was that it has increased the costs and time required to run a clinical trial within the EU.

The aims of the EU CTR are to harmonize the rules for conducting clinical trials throughout the EU and streamline the application process. A key part of the CTR is the new EU clinical trials shared central portal which will enable sponsors of trials involving multiple sites to submit a single application to the authorities of all EU Member States concerned. Applications will initially be assessed by a single reporting EU Member State and then validated by the remaining concerned Member States and undergo national ethics reviews. The new procedure is expected to accelerate trial authorization in the EU.

The EU CTR came into force in 2014 but will not apply until 6 months after the new shared central portal and database are fully functioning, currently expected to occur in March 2020. This date coincides with the United Kingdom’s time-limited implementation period (approximately 1 year) following the planned withdrawal from the EU on March 29, 2019, and is therefore expected to apply to the United Kingdom during this time. The United Kingdom will therefore have the choice to continue with the EU CTR or develop its own new regulatory framework.

It is expected that following Brexit the EMA will leave the United Kingdom and the United Kingdom will leave the European regulatory framework. The MHRA will need to expand its role in taking on new drug applications. As this may take time to implement, it will most likely result in a delay in the time it takes for the United Kingdom market to access new medicines. As the United Kingdom constitutes a smaller market for drugs than the EU (3% versus 25% of the global market) the loss of harmonized market authorization procedures could delay the availability of new medicines for British patients. For example, it has been estimated that Switzerland, which is not a member of the EMA but has a number of mutual recognition agreements with the EMA, gains access to new medicines on average 157 days later than the rest of the EU.

As to whether Brexit might provide regulatory opportunities for the United Kingdom clinical trials industry, the MHRA has taken the lead by developing a three-tiered, risk-based approach to clinical trial approval. Type A is a clinical trial with no more risk than standard medical care. It applies when a medicine is used within its market authorization, or if used off-label, there must be published evidence to justify its use. In this case, the MHRA only requires notification of the trial which will be acknowledged within 14 days, and if no objection is raised, the trial can proceed. Type B is a clinical trial with a higher risk than standard medical care; this includes dosage modifications or combinations with other medicines in which an interaction may be suspected. Type C is a clinical trial with a markedly higher risk than standard medical care.

Another opportunity for the United Kingdom clinical trials industry post-Brexit is the collection, analysis and use of “real world data,” defined as data that are collected outside the controlled constraints of conventional randomized clinical trials (RCT) to evaluate what is happening its normal clinical practice (ABPI, 2011). Randomized clinical trials are highly structured experiments or observations that have long been considered the gold standard for generating clinical data on efficacy and safety, to inform drug registration and subsequent prescribing. However, the adoption of new and innovative medicines into the marketplace requires more sophisticated evidence-based criteria that have been increasingly difficult to obtain through RCT methodology alone. As such, research methodologies and data sources that take place outside of a clinical setting are highly valued and have been said to generate real world data. Data sources can include patient registries, existing electronic health records, routinely collected administrative data, and population health surveys. Analyses from these data are increasingly playing an important role in ensuring that medicines are adopted into practice. The United Kingdom pharmaceutical industry is well placed to adapt to the growing demand for real world data to demonstrate the value of its medicines to healthcare decision-makers around the world.

It is clear that the clinical trial environment is changing and the POST findings suggest that, with its well-regarded life sciences industry, the United Kingdom could position itself as a world leader. However, the stakeholders emphasized the need for harmonized standards for both market authorization and running of clinical trials between the United Kingdom and EU.

## Brexit and Beyond: Ensuring the United Kingdom Remains a Great Place to Conduct Clinical Trials

Dr Kirsty Wydenbach (Medical Assessor at the MHRA’s Clinical Trials Unit, United Kingdom) spoke about the progress of the MHRA in working toward implementation of the new EU CTR and its strategy for the United Kingdom to continue to attract research following Brexit.

The number of clinical trials conducted in the United Kingdom has increased since 2011, with approximately 1000 clinical trials per year. In 2017, the number of Phase I trials increased by 24 applications compared with the previous year, and the number of FIH clinical trials increased by approximately 50%. However, for collaboration in joint EU clinical assessments, the number of cases in which the MHRA was nominated as the reference Member State decreased in 2017, although the MHRA is still involved in over 20% of cases. As the United Kingdom exits the EU, it is vital the United Kingdom’s ability to run trials and collaborate across the EU is maintained.

The new EU CTR is expected to apply in March 2020 during the United Kingdom’s time-limited implementation period. In the event of a “no deal” Brexit scenario, the MHRA is well-placed to implement and influence many provisions of the EU CTR. The United Kingdom Government is clear that the preferred outcome of Brexit is continued cooperation with the EU across all aspects of medicines regulation and is committed to implementing the EU CTR into United Kingdom law to the greatest possible extent. It will ensure that United Kingdom law remains aligned with parts of the EU CTR that are within MHRA control, excluding the shared portal and participation in the single assessment model.

It will still be possible for United Kingdom sponsors to run multi-state clinical trials within the EU and globally, and data generated in United Kingdom trials will be admissible to support marketing authorization applications. The MHRA will ensure the clinical trial application process is streamlined as much as possible by collaborating with partner services from the HRA, devolved administrations, ethics services, the National Institute for Health Research and the NHS.

The MHRA is piloting a scheme of combined ways of working which will test a new process resulting in a single United Kingdom decision on a clinical trial (consisting of the current ethics opinion and the MHRA clinical trial authorization) and a single clinical trial application route incorporating both the REC and the MHRA regulatory centers.

Another major change in relation to the CTR and working within the EU network will be moving away from national only assessments to harmonized assessment of multi-state trials. The MHRA is already actively involved in the Voluntary Harmonization Procedure, a coordinated prescreening of multi-national research with IMPs by competent authorities of various European Member States to identify possible serious study shortcomings prior to the official submission.

The MHRA’s goal, as set out in the Life sciences industry strategy (2017) report to the United Kingdom government, is to continue engaging with sponsors to assist with innovative protocol designs and to facilitate efficient approval of complex trials and amendments to such trials. The United Kingdom will attempt to lead innovation in clinical trial methodology, such as basket trials (where the effect of one drug is tested on a single mutation in a variety of tumor types, at the same time), and embed routine genomic analysis to make trials more targeted, smaller and more likely to deliver high efficacy.

Dr. Wydenbach concluded that the number of Clinical Trial Applications in the United Kingdom has remained steady since the United Kingdom EU referendum with a significant increase in Phase I studies, and the MHRA is committed to ensuring that the United Kingdom has the best possible environment to conduct clinical trials, both in preparation for exiting the EU and in response to the Life Sciences Industrial Strategy (Life sciences: industrial strategy, 2017).

## Conclusion

The 2018 AHPPI meeting focused on the changes in early phase drug development that have occurred over the three decades since the AHPPI was founded, the current challenges facing the United Kingdom post-Brexit, and strategies to overcome them. A wide range of exciting topics were presented and discussed including the revolution in biologics, technology and personalized medicines and the impact this has on the way patients are treated, how translational pharmaceutics is being used to ameliorate the high drug candidate failure rate, the potential of medical imaging techniques as a drug development platform and how integrated adaptive protocol designs are transforming the way that Phase I clinical trials are conducted. In closing the meeting, the AHPPI Chairman, Dr. Tim Hardman, summarized how the topics discussed demonstrated how the pharmaceutical industry is working together to ensure the United Kingdom continues to be an attractive location to conduct Phase I clinical trials.

## Author Contributions

All authors were responsible for designing the academic content of the AHHPI meeting, recruiting the various presenters, and noting the information presented at the meeting. All authors worked with the rest of the AHPPI Committee to compile the various presentations, write, and review as well as approve the manuscript.

## Conflict of Interest Statement

The authors declare that the research was conducted in the absence of any commercial or financial relationships that could be construed as a potential conflict of interest.

## Footnotes

1 http://www.ahppi.org.uk
